# Randomized controlled trial and economic evaluation of nurse-led group support for young mothers during pregnancy and the first year postpartum versus usual care

**DOI:** 10.1186/s13063-017-2259-y

**Published:** 2017-11-01

**Authors:** Jacqueline Barnes, Jane Stuart, Elizabeth Allen, Stavros Petrou, Joanna Sturgess, Jane Barlow, Geraldine Macdonald, Helen Spiby, Dipti Aistrop, Edward Melhuish, Sung Wook Kim, Diana Elbourne

**Affiliations:** 1 Department of Psychological Sciences, Birkbeck University of London, Malet Street, London, WC1E 7HX UK; 2 Department of Medical Statistics, London School of Hygiene and Tropical Medicine, London, UK; 3 Warwick Medical School, University of Warwick, Coventry, UK; 4 School for Policy Studies, University of Bristol, Bristol, UK; 5 Faculty of Medicine and Health Sciences, University of Nottingham, Nottingham, UK; 6 Sheffield Children’s NHS Foundation Trust, Sheffield, UK; 7 Department of Education, University of Oxford, Oxford, UK

**Keywords:** Early intervention, Pregnancy, Nurse, Young parenthood, Child maltreatment

## Abstract

**Background:**

Child maltreatment is a significant public health problem. Group Family Nurse Partnership (gFNP) is a new intervention for young, expectant mothers implemented successfully in pilot studies. This study was designed to determine the effectiveness and cost-effectiveness of gFNP in reducing risk factors for maltreatment with a potentially vulnerable population.

**Methods:**

A multi-site, randomized controlled, parallel-arm trial and prospective economic evaluation was conducted, with allocation via remote randomization (minimization by site, maternal age group) to gFNP or usual care. Participants were expectant mothers aged below 20 years with at least one live birth, or aged 20–24 years with no live births and with low educational qualifications. Data from maternal interviews at baseline and when infants were 2, 6 and 12 months, and video-recording at 12 months, were collected by researchers blind to allocation. Cost information came from weekly logs completed by gFNP family nurses and other service delivery data reported by participants. Primary outcomes measured at 12 months were parenting attitudes (Adult-Adolescent Parenting Index, AAPI-2) and maternal sensitivity (CARE Index). The economic evaluation was conducted from a UK NHS and personal social services perspective with cost-effectiveness expressed in terms of incremental cost per quality-adjusted life year (QALY) gained. The main analyses were intention-to-treat with additional complier average causal effects (CACE) analyses.

**Results:**

Between August 2013 and September 2014, 492 names of potential participants were received of whom 319 were eligible and 166 agreed to take part, 99 randomly assigned to receive gFNP and 67 to usual care. There were no between-arm differences in AAPI-2 total (7 · 5/10 in both, SE 0.1), difference adjusted for baseline, site and maternal age group 0 · 06 (95% CI − 0 · 15 to 0 · 28, *p* = 0 · 59) or CARE Index (intervention 4 · 0 (SE 0 · 3); control 4 · 7 (SE 0 · 4); difference adjusted for site and maternal age group − 0 · 68 (95% CI − 1 · 62 to 0 · 16, *p* = 0 · 25) scores. The probability that gFNP is cost-effective based on the QALY measure did not exceed 3%.

**Conclusions:**

The trial did not support gFNP as a means of reducing the risk of child maltreatment in this population but slow recruitment adversely affected group size and consequently delivery of the intervention.

**Trial registration:**

ISRCTN78814904. Registered on 17 May 2013.

**Electronic supplementary material:**

The online version of this article (doi:10.1186/s13063-017-2259-y) contains supplementary material, which is available to authorized users.

## Background

Recent estimates show that suboptimal parenting is a major public health issue [1]. Interventions to enhance parenting during the first year can reduce the risk of maltreatment and promote optimal child outcomes, as highlighted in UK policy documents [2, 3]. However, there is little evidence about “what works” to support vulnerable parents during pregnancy and infancy. Nurse Family Partnership (NFP), extending from pregnancy until children are 24 months old, is commonly identified from studies in the USA as a program with high-quality evidence for reducing the risk of neglect and abuse [4], although this has yet to be demonstrated in the UK [5]. Trials of NFP in the USA and the Netherlands found that it was particularly beneficial for women with “low psychological resources,” namely a combination of lower intelligence, mental health problems and low self-efficacy [6, 7].

The NFP program was introduced in the UK in 2007, renamed the Family Nurse Partnership (FNP) and offered only to first-time mothers under the age of 20 years [8]. Group FNP was developed to be offered in a group context to parents not eligible for FNP but whose children were at risk of poor outcomes, designed to use the expertise of the FNP nurses and learning from the FNP [9]. Like FNP, the group program aims to help parents develop their health, wellbeing, confidence and social support in pregnancy, their children’s health and parenting in the first year of life, and to raise aspirations about future education and employment [9]. The main difference from existing group support in the UK for expectant mothers or women with new babies, such as that offered by midwives and health visitors [10] or in Sure Start Children’s Centers [11], is that gFNP spans pregnancy and infancy, extending over 18 months and is based on a program with evidence for reducing the risk of child maltreatment. The gFNP program, delivered by two FNP family nurses (FNs) (one of whom is a practicing midwife), uses materials and the approach of the FNP program and practitioners also conduct routine antenatal care and infant health checks, based in part on the “Centering Pregnancy” program [12].

It was demonstrated in two implementation studies [13] that gFNP is acceptable to the proposed target population, potentially vulnerable mothers who are young but not eligible for FNP (aged under 20 years and expecting a second or subsequent child; aged 20 to 24 years and expecting a first child and with low educational qualifications). Following UK Medical Research Council (MRC) guidelines for evaluating complex interventions [14] the gFNP program’s impact was assessed using a randomized controlled trial (RCT).

The objective of the research was to compare families offered gFNP in early pregnancy in conjunction with usual publicly funded health and social care services with a control group receiving usual services. Outcomes were assessed when infants were 12 months old with two primary outcomes, self-reported attitudes to parenting and observations of maternal sensitivity. There were also eight secondary outcomes: at 12 months, maternal depression symptoms, maternal stress, maternal sense of competence, maternal smoking, alcohol and drug use, social support, relationship violence, observed infant cooperativeness; and breastfeeding to 6 months.

## Methods

### Study design

The study’s objective was to determine whether gFNP, compared to routine antenatal and postnatal care, could improve parenting, and hence reduce risk factors for maltreatment in a vulnerable population and also be cost-effective.

The design was a multi-site, blinded, randomized controlled, parallel-arm trial and prospective economic evaluation with eligible pregnant women at seven locations in England allocated (minimized by site and maternal age group) to one of two arms: (1) gFNP and (2) usual care. All participants (both arms) were eligible to receive usually provided publicly funded health and social care. Baseline data were collected in early pregnancy with follow-up when infants were 2 and 6 months old and the primary endpoint at 12 months.

The study was granted approval by the UK National Research Ethics Service Committee South West-Frenchay (REC reference 13/SW/0086).

### Study setting and participants

Family Nurse Partnership teams were eligible to take part in the trial if they were experienced in delivering FNP and had at least one FN who had notified their intention to practice as a midwife. Sites responded to an invitation from the FNP National Unit to take part, demonstrating that sufficient women of the relevant age and parity had given birth in the previous year, and confirming good links with community midwifery, who also signed the expression of interest. Seven sites agreed to take part, representing London (two sites), the Midlands (two sites), the North East (one site) and the North West of England (two sites).

Women eligible for the trial were expectant mothers with a gestation of 16 to 20 weeks, with expected delivery dates (EDDs) within approximately 10 weeks of each other, for each group in each site. They were either aged below 20 years at their last menstrual period (LMP) with one or more previous live births, or aged 20–24 at LMP with no previous live births and with low educational qualifications, defined as neither mathematics nor English language GCSE at grade C or higher; if both, then no more than four GCSEs at grade C or higher. They had to be able to provide consent and to speak English. Women who had previously received FNP and those with psychotic mental illness were not eligible.

### Recruitment

Full details of the recruitment can be found in the study protocol [15]. The trial commenced in February 2013, recruitment and baseline data collection commenced in July 2013, continuing to September 2014. Recruitment was in three phases, each separated by approximately 12 weeks, so that FNP teams could offer more than one group. Potential participants were identified from records by community midwives, and then after some difficulties in identifying a sufficient number [16] also by FNP midwives and Clinical Local Research Network (CLRN) research midwives, based on their age, parity and gestation from midwifery records. After written or telephone agreement for the researcher to contact the potential participant, a telephone contact confirmed eligibility by asking about educational qualifications and any psychotic mental illness. If they were eligible and interested in the study the researcher arranged a home visit so that written consent could be obtained and baseline information collected. Following baseline data collection their contact details were entered into a secure database which generated an identification number.

### Randomization and blinding

Randomization was overseen by the London School of Hygiene and Tropical Medicine Clinical Trials Unit (LSHTM CTU). The unique identification number (which included a site identifier) and age at LMP of eligible consenting mothers-to-be were passed to the central randomization service at the Health Service Research Unit (HSRU), University of Aberdeen using an automated telephone procedure. Minimization criteria (site, and age group below 20 years, 20–24 years) were used to ensure a balance of key prognostic factors. Due to low recruitment, randomization was changed from 1:1 to 2:1 in December 2013 after approval from the Trial Steering Committee (TSC), Data Monitoring Committee (DMC) and Research Ethics Committee (REC). Allocation to one of two arms was securely computer-generated and delivered by email to LSHTM which conveyed the information to study participants by post and conveyed to each gFNP team the names and contact details of women allocated to the intervention arm by fax or password-protected email, receiving confirmation of receipt by email. Participants could not be blind to allocation as they knew whether or not they had been offered gFNP, and gFNP practitioners were not blind to the intervention participants but had no knowledge of the control group. Service providers other than gFNP personnel may or may not have been blinded depending on what study participants chose to discuss when receiving usual care. The research team members responsible for collecting the questionnaire data, including the principal investigator (PI), the trial manager, the interviewers and also those scoring the videos for the CARE Index, were blind to treatment allocation.

### Procedures

*Study intervention*: Group FNP (gFNP) is designed to run from the first trimester of pregnancy until infants are 12 months old with 44 group meetings in the curriculum, 14 covering pregnancy and 30 covering infancy [17]. It is delivered to a group of women living in relatively close proximity to each other, with similar expected delivery dates (range 8–10 weeks) [9]. Meetings last around 2 h, are held in children’s centers, health centers or other suitable community facilities in the local areas served by the FNP teams. Sessions were facilitated by two experienced FNP FNs one of whom had notified their intention to practice as a midwife. The two FNs exchanged the roles of active leader (facilitating a topic and activity) and active observer, noticing behaviors and body language of members and stepping in to support the leader and maintain a positive and inclusive group environment.

The gFNP program includes content to: improve maternal health and pregnancy outcomes, improve child health and development by helping parents provide more sensitive and competent care; and to improve parental life course by helping parents develop effective support networks, plan future pregnancies, complete their education, and find employment [9]. The curriculum domains are: mother’s personal health; the maternal role; maternal life course: family and friends; environmental health; and related health and human services, with referrals made when necessary. In general all the content domains are covered to a greater or lesser extent in each of the meetings, varying according to a detailed manual. The gFNP curriculum materials and activities were modified from those used to deliver FNP to reflect group administration. They were designed to avoid a lecture context but to facilitate interaction between group members and between group members and the nurses, providing a range of engaging, often “hands-on” activities. In particular, gFNP had a particular focus on enhancing social support and social networks through dialog between group members, which is not a specific focus of home-based FNP [9, 17].

Specific to the gFNP program and following national guidelines [18], the FN midwife provided routine antenatal care during the meeting, taking an approach based on the Centering Pregnancy program [12, 19, 20] which encourages women to monitor their own health (e.g., by testing their own urine, listening to the fetal heartbeat). The Centering Pregnancy approach was perceived to correspond well with the gFNP aims in that both focus on developing self-efficacy and encouraging women to be more self-aware [9]. Once infants were born both FNs were involved in routine infant checks, conducted according to the UK National Health Service (NHS) Healthy Child Program (HCP) [10].

Appreciation of the diversity of group members is central to how the nurses deliver the content, especially for some emotive topics such as “safe relationships for our children” [9]. While there is a curriculum for each meeting the nurses were sensitive to the need for “agenda matching” related to particular issues raised; this requires the practitioners to listen to the issues that are uppermost for the group members and agree how these can be met while at the same time ensuring that the session agenda is realized and behavior adaptation is progressed for everyone. In addition to modeling of infant care, they model respectful relationships and turn-taking [21], behaviors that are expected to be of benefit to any group members with poor social skills, especially if they are experiencing difficult interpersonal relationships [9]. Study participants allocated to gFNP could also access any aspect of the HCP usual care that they wished, independently or with the guidance of the gFNP nurses.

*Control – usual care*: complete details of the care offered through the NHS to pregnant women and those with infants up to age 1 year at the time that the research was conducted can be found in the UK HCP [10 l]. The HCP, led by health visitors, is delivered through integrated services that bring together Sure Start children’s center staff, general practitioners, midwives, community nurses and others. In summary, it offers every family a program of screening tests, immunizations, developmental reviews, and information and guidance to support parenting and healthy choices. There are core universal elements provided for all families with additional progressive, preventive elements for those with medium or high risk. The universal program includes a neonatal examination, a new baby review at about 14 days, a 6- to 8-week baby examination and a review by the time the child is 1 year old and at 2 to 2.5 years old.

The aims of the HCP are to develop strong parent-child attachment and positive parenting, resulting in better social and emotional wellbeing among children; care that helps to keep children healthy and safe; healthy eating and increased activity, leading to a reduction in obesity; prevention of some serious and communicable diseases; increased rates of initiation and continuation of breastfeeding; readiness for school and improved learning; early recognition of growth disorders and risk factors for obesity; early detection of – and action to address – developmental delay, abnormalities and ill-health, and concerns about safety; identification of factors that could influence health and wellbeing in families; and better short- and long-term outcomes for children who are at risk of social exclusion.

There is a focus on supporting mothers and fathers to provide sensitive and attuned parenting, in particular during the first months and years of life. From the 12th week of pregnancy women are encouraged to see a midwife or maternity healthcare professional for a health and social care assessment of their needs, risks and choices.

### Data collection

Data collection was conducted by field researchers making four visits to participants’ homes (baseline in early pregnancy, when infants were 2 months, 6 months and 12 months old), when they administered structured questionnaires and at 12 months (if additional consent was given) they made a 3- to 5-min video-recording of the mother and infant together, presented with a standardized set of toys. Participants were given a “High Street” voucher for £20 at each home visit. Measures used at each time point are presented in Table 1 with full details in the published protocol [15]. Researchers sent completed questionnaires by post to LSHTM CTU and checks were made for receipt. Videos of play interactions were transferred by fieldworkers from camera to encrypted USB flash drives with AES 256-bit military-level security, sent by recorded delivery to the PI, with files deleted from the camera by the fieldworkers. Recordings were decrypted and saved with full anonymization of filenames on a dedicated drive separate from any other study information.

**Table 1:** Measures and data collection timetable

Measure		Baseline, pregnancy	Infant 2 months	Infant 6 months	Infant 12 months
Primary outcomes					
1.	Revised Adult-Adolescent Parenting Inventory (AAPI-2) [22]	√			√
2.	Maternal sensitivity, CARE Index [23]				√
Secondary outcomes					
1.	Infant cooperativeness, CARE Index [23]				√
2.	Maternal depression, Edinburgh Postnatal Depression Scale (EPDS) [24]	√	√	√	√
3.	Maternal stress, Parenting Stress Index, Short Form (PSI) [25]		√		√
4.	Maternal competence, Parenting Sense of Competence (PSOC) scale [26]		√		√
5.	Social support, Medical Outcomes Study (MOS) Social Support Survey [27]	√			√
6.	Maternal smoking, alcohol and drug use, questions developed for the study	√	√		√
7.	Relationship violence, questions developed for the study	√			√
8.	Breast feeding (plans and actual), questions developed for the study	√	√	√	
Economic outcomes					
1.	Maternal quality of life (EQ-5D 5 L) [28]	√	√	√	√
2.	Service use, questions developed for the study		√	√	√
Other information collected					
	Family demographic updates		√	√	√
	Baby demographics		√		
	Infant immunizations		√		√

### Outcomes

Two primary outcome measures at 12 months were used: the revised Adult-Adolescent Parenting Inventory (AAPI-2) [22], a 40-item self-report measure of attitudes to parenting previously found to discriminate between abusive and non-abusive parents [22]; and maternal sensitivity rated by observation using the CARE Index, which can range from 0 to 14 with scores of 0–4 in the risk range for child maltreatment [23]. Eight pre-specified secondary outcomes assessed infant cooperativeness [23]; maternal depression [24]; stress [25]; sense of competence [26]; social support [27]; smoking, alcohol and drug use; relationship violence; and breastfeeding to 6 months (see [15] for full details and Table 1).

To assess maternal quality-adjusted life years (QALYs) for the economic evaluation, the EuroQol EQ-5D-5 L measure [28] was administered at each time point (see Table 1). This five-dimension health status classification system can be converted to a multi-attribute utility score by applying a national tariff [29].

Adverse events were assessed using information from research midwives, maternal interview or pre-paid change of circumstances cards, and categorized as adverse (hospitalization of mother or infant other than for delivery) or severe (loss/termination of the pregnancy, congenital anomaly or birth defect, persistent or significant disability and death of either mother or infant). Severe events were reported to the REC within 15 days of the PI becoming aware of the event.

### Statistical analysis

For the individually randomized trial we proposed to recruit sufficient mothers and babies (families) to detect a difference between arms of 0 · 5 standard deviations (SDs), with 90% power at a significance level of 0 · 05 (two-tailed), considered to represent a moderate size of effect [30]. Basing calculation on the AAPI-2 [22], very conservatively assuming a correlation of 0 · 4 between pre- and post-intervention scores, at least 71 families were needed in each arm of the trial to detect this difference. Allowing for an expected 30% dropout rate (based on the first two applications of the program in England) we would need to recruit 84 families per arm of the trial. We therefore proposed, conservatively, to recruit a 100 families per arm (*N* = 200). The proposed sample size would similarly allow us to detect a change of approximately 0 · 5 SDs in the CARE Index maternal sensitivity score [23]. If this was achieved we expected to be able to detect a difference at follow-up between arms of the trial of approximately 1 · 2 with 90% power and a 5% level of significance. Based on additional calculations when changing the allocation ratio following recruitment difficulties [16], a sample size of 166 participants would have 81% power to detect an effect of 0 · 5 SDs in the primary outcomes.

Primary analyses were by intention-to-treat (ITT), i.e., based on random allocation regardless of receipt of program. Follow-up was not restricted to those receiving the program. Analyses included adjustment for baseline measure of the outcomes where available (ANCOVA) and also an “adjusted” analysis that additionally included adjustment for the minimization factors site and maternal age group. The complete case analysis provides an unbiased estimate of the ITT effect under a “missing-at-random” assumption but, in addition to the primary analyses, complier average causal effect (CACE) analyses [31] were conducted to estimate a measure of the effect of the intervention on those participants who received it as intended by the original allocation. Where outcomes were collected at multiple time points, random-effects models, using a likelihood-based approach, were simultaneously fitted to the outcomes at all the time points that they were measured so that data from all the participants contributed to the analyses, even if there were missing data at some of the follow-up points The statistical analysis plan as agreed with the DMC in December 2014 was for the primary analysis to exclude all data outside pre-specified windows, i.e., after 12 months plus 60 days, 6 months plus 45 days and 2 months plus 30 days. However, in addition, sensitivity analyses were conducted with all data including those outside the windows. For the primary outcome of the AAPI-2 [22] a linear regression model was used to estimate a mean difference in 12-month AAPI-2 scores between the two arms of the trial. For the primary outcome maternal sensitivity score [23] a mixed-effects model was planned with a random effect at the mother level (to allow for multiple births) to estimate a mean difference in maternal sensitivity score between the two arms of the trial. However, only one set of twins was available for this analysis and their responses were identical. Therefore, it was not possible to include a random effect and the analysis was carried out at the mother level using a linear regression model.

For the secondary outcomes, appropriate generalized linear models were used to examine the effect of the intervention. Odds ratios and mean differences are reported with 95% confidence intervals (CIs). As for the primary outcomes, where continuous measures were available at baseline they were adjusted for in the analysis with additional analyses adjusted for site and maternal age group. Where there was evidence of non-normality in the continuous outcome measures, non-parametric bootstrapping with 1000 samples was used to estimate the effect of the intervention and bias-corrected CIs are reported [32]. Where this was done, *p* values were estimated using permutation tests. Statistical analyses were conducted using Stata version 14. An independent Data Monitoring Committee (DMC) oversaw the trial.

### Economic evaluation

A within-trial economic evaluation was conducted from a UK NHS and personal social services perspective. Primary research methods estimated the costs of delivering gFNP, including development and training of accredited providers, the cost of delivering the group sessions, participant monitoring activities, and any follow-up/management. This primarily involved asking each of the gFNP practitioners in each site to prospectively complete detailed weekly activity logs outlining the cost of delivering each gFNP session, including costs associated with preparation time, program delivery time, indirect administrative activities, home visits and telephone contacts, as well as gFNP-related training and supervision activities. The weekly activity logs also recorded the mode, distance and time spent traveling by each practitioner as a result of gFNP-related activities. They also recorded additional expenditures associated with refreshments, materials, cards or gifts, participant travel, partner travel, child-care costs and miscellaneous expenditures associated with weekly gFNP-related activities. The costs of venue hire were estimated separately within each site. The total costs of delivering the gFNP program across each group and site were subsequently converted into group and site-specific estimates of average cost per session per attending woman using separately collected attendance data for each group within each site. Broader resource utilization was captured through maternal questionnaires completed at each follow-up point and provided profiles of hospital and community health and social services received by each trial participant (and, in the case of the postpartum questionnaires, their baby). Information was also collected regarding use of legal services and costs borne by the trial participants or their family members or friends as a result of the trial participants’ (and, in the case of the postpartum questionnaires, their baby’s) health status, over the relevant time horizons. Unit costs (£, 2014–2015 prices) were collected from national sources in accordance with guidelines and attached to resource use. Health utilities generated from EuroQol EQ-5D-5 L [28] responses were used to estimate QALY profiles for each woman, calculated as area under the baseline-adjusted utility curve, assuming linear interpolation between utility measurements. Costs and QALYs accrued by each trial participant beyond the first 12 months of follow-up were discounted at 3 · 5% as recommended by the National Institute for Health and Care Excellence (NICE) [33]. Multiple imputation using chained equations [34] and predicted mean matching was carried out on the EQ-5D-5 L, as well as cost estimates, at 2, 6 and 12 months postpartum [35]. Mean (standard error (SE)) costs by cost category and mean (SE) total costs were estimated by trial allocation arm for all time periods.

Cost-effectiveness results are reported as incremental cost-effectiveness ratios (ICERs), calculated as the difference in mean costs divided by the difference in mean outcomes (QALYs or maltreatment outcome measures) between the trial comparators. The nonparametric bootstrapping approach was used to determine the level of sampling uncertainty surrounding the mean ICERs by generating 10,000 estimates of incremental costs and benefits, represented graphically on four quadrant cost-effectiveness planes. Cost-effectiveness acceptability curves (CEACs) showing the probability that the gFNP program is cost-effective relative to standard care across a range of cost-effectiveness thresholds were also generated based on the proportion of bootstrap replicates with positive incremental net benefits. Several sensitivity analyses were undertaken to assess the impact of uncertainty surrounding components of the economic evaluation. These included: (1) adopting a wider societal perspective, (2) restricting the analyses to complete cases, and (3) recalculating the average cost per gFNP session per attending woman by varying (1) the mean number of gFNP sessions attended to the highest and lowest observed mean values across all groups across all sites and (2) the number of gFNP group participants to the highest and lowest number of observed values across all groups across all sites. Subgroup analyses were conducted for the main cost-effectiveness results by: (1) program completers (no, yes) where women who participated in a pre-specified number of group sessions (at least 17 sessions) were regarded as “program completers” and (2) program phase (1, 2, 3) to test whether organizational learning may have influenced the cost-effectiveness of the gFNP program. Analyses for the economic evaluation were conducted using Stata software version 14.

## Results

**Fig. 1:**
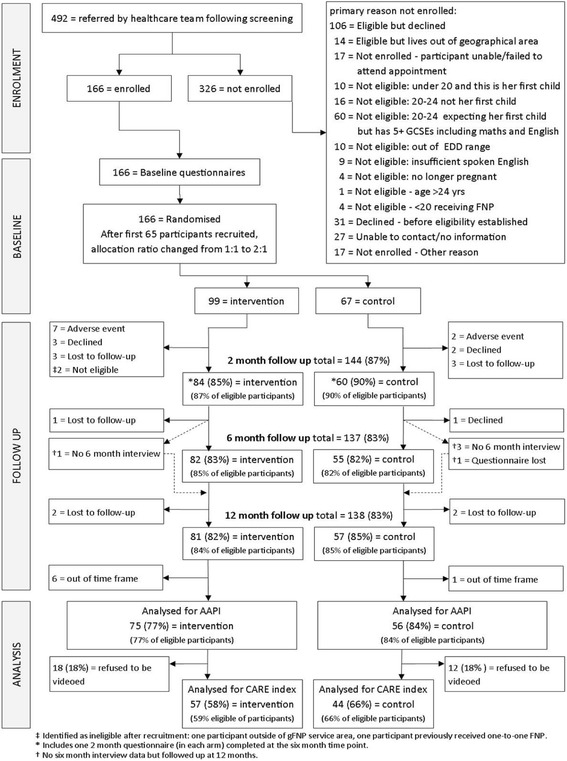
Trial consolidated standards of reporting trials (CONSORT) diagram

Between 22 August 2013 and 17 September 2014, 492 names of potential participants were received; 286 were definitely eligible, and 166 agreed to participate with 99 randomly assigned to receive gFNP and 67 to usual care (see Fig. 1). After recruitment, two women in the intervention arm were found to be ineligible (one was outside the FNP service area, one had received FNP). All follow-up data were retrieved by February 2016. Participants in the two randomized arms appear comparable at baseline in terms of their demographic characteristics (see Table 2). Although information from follow-up at around 2 months postpartum was collected for 144 participants (84 intervention and 60 control), 16 (nine intervention, seven control) were out of the agreed time window, leaving 128 (75 intervention, 53 control). From the follow-up at around 6 months postpartum information was collected for 137 participants (82 intervention and 55 control); however, 16 (12 intervention, four control) were out of the agreed time window leaving 121 (70 intervention, 51 control) (Fig. 1). Although 138 12-month interviews were carried out (81 intervention, 57 control), seven (six intervention, one control) were out of the agreed time window, leaving 131 (75 intervention, 56 control) eligible for the primary analysis. The primary analysis for the CARE Index (co-primary outcome) was based on 101 videos (57 intervention, 44 control) (see Fig. 1).

**Table 2:** Sociodemographic characteristics at baseline

	gFNP (*N* = 97)	Usual care (*N* = 67)
Age at last menstrual period, mean (SD)	21 · 0 (1 · 8)	21 · 2 (1 · 8)
Educational qualifications – GCSEs or equivalent?		
Yes	73 (75 · 3%)	55 (82 · 1%)
No	24 (24 · 7%)	12 (17 · 9%)
GCSEs, mean (SD)	6 · 7 (3 · 1) *n = 70*	6 · 4 (2 · 7) *n = 54*
GCSEs, grade C or higher, mean (SD)	3 · 8 (3 · 6) *n = 69*	3 (2 · 5) *n = 53*
Educational qualifications – other?		
Yes	79 (82 · 3%)	56 (83 · 6%)
No	17 (17 · 7%)	11 (16 · 4%)
Ethnicity		
White – British	61 (63 · 5%)	48 (71 · 6%)
White – Irish	2 (2 · 1%)	0 (0 · 0%)
Any other White background	2 (2 · 1%)	3 (4 · 5%)
Asian British – Pakistani	5 (5 · 2%)	5 (7 · 5%)
Asian British – Bangladeshi	1 (1 · 0%)	0 (0 · 0%)
Black British – Caribbean	14 (14 · 6%)	6 (9 · 0%)
Black British – African	3 (3 · 1%)	2 (3 · 0%)
Mixed	8 (8 · 3%)	3 (4 · 5%)
Current partner?		
Yes	83 (85 · 6%)	59 (88 · 1%)
No	14 (14 · 4%)	8 (11 · 9%)
Current partner biological father of expected child?		
Yes	83 (100%)	59 (100%)
Marital status		
Married	10 (10 · 4%)	8 (11 · 9%)
Unmarried/co-habiting	43 (44 · 8%)	37 (55 · 2%)
Single	43 (44 · 8%)	22 (32 · 8%)
Number of people currently living with, mean (SD)	2 · 9 (1 · 5) *n = 96*	3 · 1 (1 · 6)
Current household membership		
Own mother/parents	11 (11 · 7%)	7 (10 · 9%)
Husband/partner	24 (25 · 5%)	24 (37 · 5%)
Husband/partner and others (not own mother)	10 (10 · 6%)	6 (9 · 4%)
Own mother/parents and others, not husband/partner	14 (14 · 9%)	10 (15 · 6%)
Own mother/parents and others, with husband/partner	6 (6 · 4%)	5 (7 · 8%)
Husband/partner and others	2 (2 · 1%)	3 (4 · 7%)
Other adults	12 (12 · 8%)	6 (9 · 4%)
Lives alone	15 (16 · 0%)	3 (4 · 7%)
Type of accommodation		
House or bungalow	68 (70 · 1%)	49 (73 · 1%)
Flat, low-rise	12 (12 · 4%)	5 (7 · 5%)
Flat, high-rise, first 3 floors	5 (5 · 2%)	12 (17 · 9%)
Flat, high-rise, above 3rd floor	4 (4 · 1%)	0 (0 · 0%)
Room or bedsit	2 (2 · 1%)	1 (1 · 5%)
Hostel	2 (2 · 1%)	0 (0 · 0%)
Supported housing	1 (1 · 0%)	0 (0 · 0%)
Group home/shelter	2 (2 · 1%)	0 (0 · 0%)
Other	1 (1 · 0%)	0 (0 · 0%)
Enrolled in any school or educational program?		
Yes	12 (12 · 4%)	9 (13 · 4%)
No	85 (87 · 6%)	58 (86 · 6%)
Course details		
School, up to year 11	1 (8 · 3%)	0 (0 · 0%)
School, year 12 or 13 or 6th form college	1 (8 · 3%)	0 (0 · 0%)
Access course	1 (8 · 3%)	1 (11 · 1%)
Vocational course	6 (50 · 0%)	2 (22 · 2%)
University	3 (25 · 0%)	6 (66 · 7%)
Ever employed?		
Yes	76 (78 · 4%)	56 (83 · 6%)
No	21 (21 · 7%)	11 (16 · 4%)
Currently employed?		
Yes, full-time	30 (39 · 5%)	28 (50 · 0%)
Yes, part-time	14 (18 · 4%)	8 (14 · 3%)
No	32 (42 · 1%)	20 (35 · 7%)

*GCSE* General Certificate of Education, taken usually at age 15/16 years; generally required before any further educational course can be taken, *gFNP* Group Family Nurse Partnership, *SD* standard deviation

Miscarriage or termination in early pregnancy, before the time that participants could begin attending gFNP sessions, was identified for five of the intervention-arm participants, with one other suspected but not confirmed, and for one control arm member. There was one late loss of pregnancy in the eighth month for an intervention participant and one infant death occurred at 3 months for a member of the control arm (see Fig. 1).

The 97 trial participants in the intervention arm were allocated to 16 planned groups; five sites planned for two groups and two sites planed for three. In addition, one participant attended sessions offered in both groups as the first group was terminated prematurely. Overall, the 97 trial participants in the intervention arm attended a mean of 10 · 3 sessions (SD 13 · 4) but a substantial proportion (39, 40%) did not attend any sessions. Of the 97, 17 were never allocated a gFNP ID number by the relevant gFNP team. Reasons were: six were contacted and refused gFNP; three were contacted and agreed but did not attend any sessions; one miscarried by the time the team contacted them; one moved away; two were not contactable; with no information provided for four. Twenty-two of the remaining 80 registered for gFNP did not attend any sessions, 10 of whom were allocated to groups that did not offer any sessions due to low enrollment. Five of those were offered FNP and others were referred back to existing services. Thus, of the 97 allocated to the intervention arm, 58 took part in at least one gFNP session.

There was no suggestion of an important effect of gFNP on either of the two primary outcomes in the ITT analysis based on outcomes available within the agreed time frame (AAPI-2, *N* = 131, CARE Index *N* = 101): the AAPI-2 [22] total was 7 · 5/10 (SE 0 · 1) in both arms (difference adjusted for baseline, site and maternal age group 0 · 06; see Additional file 1: Table S1 for baseline) (95% CI − 0 · 15 to 0 · 28, *p* = 0 · 59); and mother’s sensitivity using the CARE Index [23] was mean 4 · 0 in the intervention arm (SE 0 · 3) and 4 · 7 in the control arm (SE 0 · 4) (difference adjusted for site and maternal age group − 0 · 68, 95% CI − 1 · 62 to 0 · 16, *p* = 0 · 25) (see Table 3).

**Table 3:** Primary and secondary outcomes and estimated Group Family Nurse Partnership (gFNP) intervention effects at 12 months

Measure	gFNP	Usual care	Unadjusted effect estimate^a^		Adjusted effect estimate^b^	
	Mean (SE)	Mean (SE)	Difference (95% CI)	*p* value	Difference (95% CI)	*p* value
AAPI-R	*N* = 75	*N* = 56				
Total (/10)	7 · 5 (0 · 1)	7 · 5 (0 · 1)	0 · 05 (−0 · 17, 0 · 24)	0 · 68	0 · 06 (− 0 · 15, 0 · 28)	0 · 59
CARE Index	*N* = 57	*N* = 44				
Mother’s sensitivity (/14)	4 · 0 (0 · 3)	4 · 7 (0 · 4)	− 0 · 76 (− 1 · 68, 0 · 13)	0 · 22	− 0 · 68 (− 1 · 62, 0 · 16)	0 · 25
Infant cooperativeness (/14)	3 · 0 (0 · 3)	3 · 5 (0 · 3)	− 0 · 49 (− 1 · 25, 0 · 34)	0 · 38	− 0 · 45 (− 1 · 25, 0 · 33)	0 · 42
Depression, EPDS	*N* = 83	*N* = 59				
Total (/30)	3 · 8 (0 · 5)	4 · 1 (0 · 6)	− 0 · 07 (− 0 · 76, 0 · 62)	0 · 85	0 · 05 (− 0 · 68, 0 · 77)	0 · 90
Parenting stress, PSI	*N* = 83	*N* = 58			*N* = 83	*N* = 58
Total (/180)	73 · 4 (1 · 5)	74 · 9 (2 · 0)	− 0 · 97 (− 3.65, 1.70)	Total (/180)	73 · 4 (1 5)	74 · 9 (2 · 0)
Parenting competence (PSOC)	*N* = 81	*N* = 58				
Total (/102)	60 · 9 (0 · 4)	60 · 7 (0 · 6)	− 0 · 12 (− 0 · 92, 0 · 67)	0 · 76	− 0 · 18 (− 1.03, 0 · 67)	0 · 68
Social support, MOS	*N* = 73	*N* = 55				
Total (/100)	84 · 6 (2 · 2)	84 · 5 (2 · 3)	− 0 · 59 (− 5 · 71, 4 · 53)	0 · 82	− 0 · 45 (− 5.45, 4 · 59)	0 · 85
Relationships, abuse	*N* = 75	*N* = 56				
Total (/6)	0 · 4 (0 · 1)	0 · 5 (0 · 1)	− 0 · 07 (− 0 · 39, 0 · 19)	0 · 63	− 0 · 10 (− 0 · 40, 0 · 17)	0 · 47
Smoking, alcohol, drug use	*N* = 75	*N* = 56				
Total (/24)	17 (0 · 3)	16 · 6 (0 · 3)	− 0 · 2 (− 1 · 19, 0 · 79)	0 · 71	− 0 · 20 (− 1 · 16, 0 · 82)	0 · 70
Breastfeeding at 6 months?	*N* = 70	*N* = 51				
Yes	15 (21 · 4)	4 (7 · 8)	3 · 2 (0 · 99, 10 · 3)	0 · 05	3 · 46 (1.02, 11.75)	0 · 05
No	55 (78 · 6)	47 (92 · 2)	1			

^a^Analysis of covariance – adjusted for baseline where possible

^b^Adjusted where possible for baseline, and for site and maternal age group

Note: *CI* confidence interval, *AAPI-2* Revised Adult-Adolescent Parenting Inventory, *EPDS* Edinburgh Postnatal Depression Scale, *PSI* Parenting Stress Index, *PSOC* Parenting Sense of Competence, *MOS* Medical Outcomes Study Social Support Survey, *SE* standard error

Using a complier average causal effect (CACE) analysis [31] to take account of compliance made very little difference to the ITT results for the AAPI-2 either when compliance was defined as attending at least one group session (difference 0 · 13, 95% CI − 0 · 41 to 0 · 69, *p* = 0 · 64) or when compliance was defined as attending at least 17 group sessions (difference 0 · 17, 95% CI − 0 · 91 to 1 · 24, *p* = 0 · 76). The corresponding results for mother’s sensitivity in the CARE Index are difference − 1 · 29, 95% CI − 2 · 78 to 0 · 19, *p* = 0 · 09 when compliance was defined as attending at least one group session, and difference − 2 · 61, 95% CI − 5 · 57 to 0 · 35, *p* = 0 · 08 when compliance was defined as attending at least 17 group sessions (see Table 4).

**Table 4:** Primary outcomes and estimated intervention effects at 12 months − complier average causal effect estimates

Measure	gFNP *N* = 75	Usual care *N* = 56	Unadjusted effect estimate^a^ Difference (95% CI)	*p*
	Mean (SE)	Mean (SE)		
Adult-Adolescent Parenting Inventory-2 [22]				
Total (/10) attended at least 1 session	7 · 6 (0 · 2)	7 · 4 (0 · 2)	0 · 13 (− 0 · 41, 0 · 69)	0 · 64
Total (/10) attended at least 17 sessions	7 · 9 (0 · 2)	7 · 8 (0 · 5)	0 · 17 (− 0 · 91, 1 · 24)	0 · 76
CARE Index maternal sensitivity [23]				
Total (/10) attended at least 1 session	4 · 1 (0 · 3)	5 · 4 (0 · 7)	− 1 · 29 (− 2 · 78, 0 · 19)	0 · 09
Total (/10) attended at least 17 sessions	4 3 (0 · 5)	6 · 4 (1 · 5)	− 2 · 61 (− 5 · 57, 0 · 35)	0 · 08

^a^Analysis of covariance – (adjusted for baseline)

Note: the numbers in the control group column are the means of the sample of the controls that would have expected to have been compliers had they received the intervention

*CI* confidence interval, *gFNP* group Family Nurse Partnership, *SE* standard error

There is no evidence of any effect of the intervention on any of the eight pre-specified secondary outcomes (see Table 3) with the exception that the proportion of women still breastfeeding at 6 months is higher in the intervention arm (adjusted OR 3 · 2 (0 · 99, 10 · 6); *p* = 0 · 05). The sensitivity analyses including all participants irrespective of whether they were within the pre-specified time window (see Additional file 1: Table S2) supported the findings of the primary and secondary analyses.

**Fig. 2:**
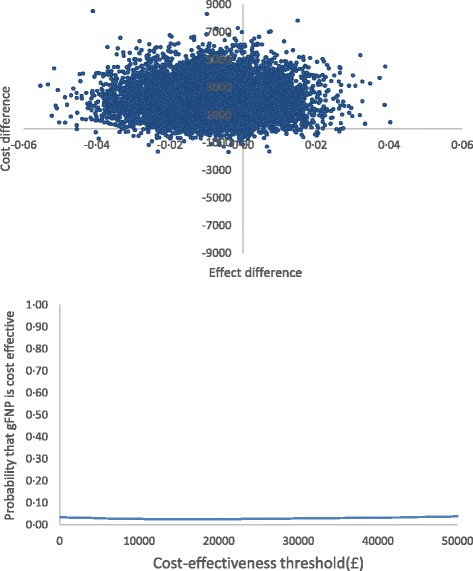
Cost-effectiveness plane and cost-effectiveness acceptability curve based upon the quality-adjusted life year (QALY) outcome: imputed data, National Health Service (NHS) and personal social services (PSS) perspective (£, 2014–2015 prices)

Economic costs for women with complete data are presented in the Additional file 1: Table S3, by trial arm, study period and cost category. Adopting a study perspective of the NHS and personal social services (PSS) and measuring health outcomes in terms of QALYs, the average total cost was £8179 in the gFNP intervention arm, compared with £6107 in the control arm, generating a mean incremental cost of £2072. The mean incremental cost-effectiveness of gFNP was estimated at − £247,485 per QALY gained, i.e., on average the intervention was associated with a net positive cost and a net negative effect (see Table 5). Regardless of the value of the cost-effectiveness threshold, the probability that gFNP is cost-effective does not exceed 3% (see Fig. 2). This pattern of results was broadly replicated when outcomes were measured using the CARE Index maternal sensitivity [23]. It is notable, however, that when outcomes were measured in terms of *change* in AAPI-2 [22] score between baseline and 12 months postpartum, the gFNP intervention was associated with a positive health effect (mean incremental gain in AAPI-2 score 0 · 02). For this outcome measure, the probability that gFNP is cost-effective reached 25 · 1% at a notional £20,000 cost-effectiveness threshold.

**Table 5:** Baseline cost-effectiveness based upon quality-adjusted life year (QALY) and primary outcomes: imputed data, NHS and PSS perspective^a^

Outcome Measure	Mean costs (95% CI)			Mean effects (95% CI)				Probability that gFNP is				
	gFNP (£)	Usual care (£)	Difference (£)	gFNP	Usual care	Difference	ICER (£)	More effective^b^ (%)	Less costly^b^ (%)	Cost-effective^b^ (%)^#^	Cost-effective^b^ (%)^±^	Cost-effective^b^ (%)^∞^
QALY [28]	*N* = 97	*N* = 67		*N* = 97	*N* = 67							
	8179 (5397, 10961)	6107 (5029, 7184)	2072 (− 843, 4988)	0 · 92 (0 · 84, 1 · 00)	0 · 93 (0 · 85, 1 · 00)	− 0 · 01 (− 0 · 05, 0 · 03)	− 247,485 (NW)	19 · 2	2 · 8	2 · 0	2 · 3	3 · 0
AAPI-2 [22]	*N* = 97	*N* = 67		*N* = 97	*N* = 67							
	8179 (5903, 10455)	6107 (5160, 7054)	2072 (− 392, 4537)	0 · 27 (0 · 14, 0 · 40)	0 · 25 (0 · 12, 0 · 38)	0 · 02 (− 0 · 17, 0 · 21)	111,334 (NE)	58 · 4	1 · 9	19 · 1	25 · 1	32 · 9
CARE Index maternal sensitivity [23]	*N* = 97	*N* = 67		*N* = 97	*N* = 67							
	8179 (5903, 10455)	6107 (5160, 7054)	2072 (− 392, 4537)	3 · 97 (3 · 54, 4 · 39)	4 · 84 (4 · 30, 5 · 38)	− 0 · 87 (− 1 · 55, − 0 · 19)	− 2382 (NW)	1 · 2	1 · 4	< 1	< 1	< 1

^a^(£, 2014–2015 prices), ^b^Based on 10,000 bootstrap replicates of the dataset

The gFNP intervention was considered to be “cost-effective” if it had positive net benefit at a: ^#^GBP £15,000 cost-effectiveness threshold, ^±^GBP £20,000 cost-effectiveness threshold, ^∞^GBP £30,000 cost-effectiveness threshold

*gFNP* Group Family Nurse Partnership, *GBP*, pounds sterling, *NHS* National Health Service, *PSS* personal social services, *CI* confidence interval, *ICER* incremental cost-effectiveness ratio, *NW* north-west quadrant of cost-effectiveness plane, *NE* north-east quadrant of the cost-effectiveness plane, *QALY* quality-adjusted life years, *AAPI-2* Adult-Adolescent Parenting Inventory

Sensitivity analyses were also conducted on the economic data. Broadening the study perspective to that of society as a whole had little effect on the results. In particular, when the QALY metric was adopted as the primary outcome measure, the mean ICER remained in the north-west quadrant of the cost-effectiveness plane and the probability that gFNP is cost effective at a £20,000 cost-effectiveness threshold remained at 2 · 5%. Increasing the mean number of gFNP sessions attended to the highest number of sessions observed across all groups across all sites and increasing the number of gFNP group participants to the highest number of participants observed across all groups across all sites had the effect of decreasing the mean cost difference between the trial arms. Nevertheless, the mean ICER for gFNP remained in the north-west quadrant of the cost-effectiveness plane, and the probability of cost-effectiveness for the intervention did not exceed 20% at a £20,000 cost-effectiveness threshold. Pre-specified subgroup analyses revealed no evidence that either program completion or the program phase had a positive effect on the cost-effectiveness of the gFNP program (see Additional file 1: Tables S4 to S7).

## Discussion

Neither the main intention-to-treat analyses nor the CACE analysis, in which comparisons focused on mothers who had attended either at least one session or at least 17, identified any evidence that the gFNP program, compared to routine antenatal and postnatal services, could reduce risk factors for maltreatment based on mothers’ attitudes to parenting and sensitive behavior when interacting with their 1-year-old infants. Only one of eight secondary outcomes showing evidence of a positive impact of gFNP (women in the intervention arm of the trial were more likely to breastfeed their baby up to 6 months) which aligns with the World Health Organization’s (WHO) recommendations [36], and may also have some potential for enhancing other parenting behaviors such as maternal sensitivity [37]. Overall, and contrary to the prediction, this study did not show that the gFNP program is more effective than usual care for reducing the likelihood of child maltreatment with this population of young and potentially vulnerable mothers, and the economic evaluation demonstrated that it is more costly than treatment as usual. This reflects the findings of a recent UK trial of the related home delivered FNP program which also did not identify any impact on the likelihood of child maltreatment, although secondary outcomes related to child language were positive [5].

However, the overall results need to be considered in the context of the threat to external validity due to slow recruitment [16], which affected the size of groups, the extent of attrition and, in some cases, whether or not a sufficient number were recruited to run a group at all. Many months of preparation notwithstanding, it proved challenging to identify potential participants, the main consequence being that almost all the groups delivered were suboptimal in terms of the number of clients, meaning that dynamic exchange between group members in terms of beliefs or parenting practices, and between group members and the FNs facilitating the groups, may have been reduced. The groups delivered in pilot work were larger [13] and many participants perceived substantial benefits, in particular in terms of one of the secondary outcomes in this study, social support, which nevertheless was not shown to be affected by gFNP in this trial. Retention at trial follow-up was good, which reinforces the view that initial identification of participants [16] was the primary reason for difficulties in setting up and maintaining groups.

The Dutch trial of FNP [7] offered it to a population with several vulnerabilities. Increasing the vulnerability criteria for eligibility to gFNP might have increased the potential of the study to identify an impact, but to establish groups of sufficient size with women who live in close proximity to each other and also to have similar due dates, would require fewer and not more eligibility requirements which would make evaluation a challenge. Basing eligibility on more criteria would also require more information to be available in antenatal records. Changes in recording systems and restricted access for primary care and maternity care could mean that the identification of potential participants with more risk factors would be challenging, reflecting the difficulties faced in this trial [16].

Implementation studies of gFNP found that program delivery could be sustained if groups started with between eight and twelve members [13]. A number of perceived impacts were also identified, though they mainly focused on increased social support, the capacity to manage relationships successfully, and maternal confidence [13, 17]. These were examined as secondary outcomes in this trial but did not show any difference from women assigned to usual care, which suggests that the UK Healthy Child Program [10] may provide families with sufficient support in terms of these outcomes. Nevertheless it is worth noting that mothers in both arms of the trial had below average CARE Index sensitivity scores placing them only just above the high-risk range (0–4) [23]. Thus, these young women, relatively new or new to parenting, may have benefitted from a group intervention that focuses specifically on enhancing playful, stimulating and sensitive mother-infant activities, such as the Australian Community HUGS program [38]. Alternatively, while group support starting in pregnancy may help with building social capital, specific parenting practices or attitudes may be best enhanced with postnatal programs such as video-interaction guidance [39] or parent-infant psychotherapy [40].

## Conclusions

In conclusion, it has been shown in RCTs in the USA [6], and in Europe [7], though not in England [5], that a home-based, nurse-delivered program, Nurse Family Partnership (NFP, in the UK Family Nurse Partnership, FNP) starting in pregnancy and continuing until infants are 24 months, reduces the likelihood of child maltreatment when offered to potentially vulnerable, young, first-time mothers. The gFNP program was developed prior to the publication of the UK trial of FNP to have similar objectives. In retrospect, if the UK trial results had been available, the development of gFNP might have focused more on sensitive parenting and child protection in the knowledge of the minimal impact in the UK of one-to-one FNP on outcomes indicative of a risk for maltreatment, or maltreatment itself.

Implementation evaluations of gFNP found that it was acceptable to the target population and to the nurses delivering the program with perceived positive benefits, such as greater parenting competence, though much of the impact was thought to be in relation to social support and infant development [13]. This RCT study has shown, however, that the gFNP program with this UK population is not a cost-effective means of supporting parenting and reducing the risk of child maltreatment in comparison with existing universal services. Further development and refinement of the program is recommended prior to any subsequent implementation or evaluation. Finally, any subsequent evaluation of gFNP will need strong involvement of local midwifery teams to identify potential participants in early pregnancy as efficiently as possible.

## Additional file

Additional file 1: Baseline information and results of additional analyses. Baseline values for questionnaires. Sensitivity analyses of primary and secondary outcomes with all participants, details of economic costs of service use for cases with complete data by trial allocation, cost-effectiveness results based upon the QALY and maltreatment outcomes by societal perspective and by NHS and PSS perspectives, economic sensitivity analysis that varied gFNP session attendance and group size, and economic subgroup analyses by phase of recruitment. (DOCX 59 kb)

## Abbreviations

CI: Confidence interval
CLRN: Clinical local research network
CTU: Clinical trials unit
DMC: Data Monitoring Committee
EDD: Expected delivery date
FN: Family nurse, according to USA NFP National Office guidelines
FNP: Family Nurse Partnership home-visiting program (UK)
GBP: Pounds sterling
GCSE: General Certificate of Secondary Education
gFNP: Group-based Family Nurse Partnership
HSRU: Health Services Research Unit
ISRCTN: International standard randomized controlled trial number
LMP: Last menstrual period
LSHTM: London School of Hygiene and Tropical Medicine
MOS: Medical outcomes study
NFP: Nurse Family Partnership, renamed FNP in the UK
NHS: UK National Health Service
NICE: UK National Institute for Health and Clinical Excellence
NIHR: UK NHS National Institute for Health Research
PI: Principal Investigator
PSS: Personal social services
QALY: Quality-adjusted life year
RCT: Randomized controlled trial
SD: Standard deviation
SE: Standard error
TMG: Trial management group
TSC: Trial steering committee
UK: United Kingdom
