# Supplementary material for: Randomized controlled trial and economic evaluation of nurse-led group support for young mothers during pregnancy and the first year postpartum versus usual care
Source: Trials. 2017 Nov 1;18:508. doi: 10.1186/s13063-017-2259-y (PMC5667036; doi:10.1186/s13063-017-2259-y)
Supplement: Additional file 1: — Baseline information and results of additional analyses. Baseline values for questionnaires. Sensitivity analyses of primary and secondary outcomes with all participants, details of economic costs of service use for cases with complete data by trial allocation, cost-effectiveness results based upon the QALY and maltreatment outcomes by societal perspective and by NHS and PSS perspectives, economic sensitivity analysis that varied gFNP session attendance and group size, and economic subgroup analyses by phase of recruitment. (DOCX 59 kb) [file 13063_2017_2259_MOESM1_ESM.docx]

**Additional tables**

**Table A1 Baseline scores for questionnaires**

| **Category** | **Intervention (N=97)** | **Control (N=67)** |
| --- | --- | --- |
|  | Mean (SD) | Mean (SD) |
| **Adult Adolescent Parenting Inventory – Revised (higher - positive) [22]** | | |
| Total (/10)  *missing* | 7·2 (0·8)  *n=9* | 7·2 (0·9)  *n=2* |
| **Edinburgh Postnatal Depression Scale (higher - more depressed) [24]** | | |
| Total (/30)  *missing* | 6·9 (4·7)  *n=1* | 7·7 (5·0)  *n=1* |
| **Social Networks (higher - more support) [27]** | | |
| Total (/100)  *missing* | 85·8 (15·6)  *n=2* | 85·3 (16·4) |
| **Relationships (higher – more abuse)** | | |
| Total abuse (/8) | 0·6 (0·9) | 0·5 (0·8) |

**Table A2: Sensitivity analyses with all participants**

| **Measure** | **gFNP** | **Usual care** | **Unadjusted Effect Estimate^1^** | | **Adjusted  Effect Estimate^2^** | |
| --- | --- | --- | --- | --- | --- | --- |
|  | **Mean (SE)** | **Mean (SE)** | **Difference  (95% CI)** | **p-value** | **Difference  (95% CI)** | **p-value** |
| **AAPI-2 [22]** | N=81 | N=57 |  | | | |
| Total (/10) | 7·5 (0·1) | 7·5 (0·1) | 0·02 (-0·19, 0·23) | 0·83 | 0·02 (-0·18, 0·24) | 0·83 |
| **CARE Index [23]** | N=61 | N=44 |  | | | |
| Maternal sensitivity (/14) | 3·8 (0·3) | 4·7 (0·4) | -0·84 (-1·71, 0·07) | 0·13 | -0·73 (-1·60, 0·12) | 0·18 |
| Infant cooperativeness (/14) | 2·9 (0·3) | 3·5 (0·3) | -0·54 (-1·31, 0·21) | 0·30 | -0·47 (-1·27, 0·26) | 0·36 |
| **EPDS [24]** | N=83 | N=59 |  | | | |
| 12 months, total (/30) | 3·8 (0·5) | 4·1 (0·6) | -0·12 (-0·48, 0·73) | 0·6 | -0·03 (-0·63, 0·57) | 0·92 |
| **PSOC [26]** | N=81 | N=57 |  | | | |
| 12 months, total (/102) | 60·9 (0·4) | 60·7 (0·6) | 0·12 (-0·48, 0·73) | 0·68 | 0·08 (-0·76, 0·91) | 0·86 |
| **PSI stress[25]** | N=81 | N=59 |  | | | |
| 12 months, total (/180) | 73·4 (1·5) | 74·9 (2·0) | -0·72 (-3.25, 1.80) | 0·56 | -0·89 (-3.46, 1.68) | 0·50 |
| **MOS social support[27]** | N=81 | N=57 |  | | | |
| 12 months, total (/100) | 85·1 (2·0) | 84·6 (2·3) | -0·03 (-5·09, 5·08) | 0·99 | 0·07 (-5·18, 5·00) | 0.98 |
| **Relationship abuse** | N=81 | N=57 |  | | | |
| 12 months, total (/6) | 0·4 (0·1) | 0·5 (0·1) | -0·06 (-0·37, 0·21) | 0·66 | -0·08 (-0·37, 0·21) | 0·55 |
| **Smoking, alcohol and drugs** | N=81 | n=57 |  | | | |
| 12 months, total (/24) | 3·1 (0·3) | 3·2 (0·4) | -0·12 (-1·07, 0·90) | 0·83 | -0·09 (-1·02, 0·95) | 0·86 |
| **Still breastfeeding at six months** | N=82 | N=55 |  |  |  |  |
| Yes | 16 (19·5) | 4 (7·3) | 3·1 (0·97, 9·81) | 0·06 | 3·51 (1.05, 11.69) | 0·05 |
| No | 66 (80·5) | 51 (92·7) | 1 |  |  |  |

^1^ Analysis of covariance – adjusted for baseline where possible

^2^ Adjusted where possible for baseline and for site and maternal age group

**Table A3: Economic costs for cases with complete data by trial allocation, study period and cost category (£, 2014-15 prices)**

| **Cost category by period** | **gFNP**  **Mean (SE) Cost** | **Usual care**  **Mean (SE) Cost** | **Mean difference** | ***P* value^a^** | **Bootstrap 95% CI^b^** |
| --- | --- | --- | --- | --- | --- |
| **Baseline to 2 months (n=141 total; n=82 intervention and n=59 control)** | | |  |  |  |
| Mother: delivery costs | 1922·6 (114·0) | 1871·7 (118·7) | 50·9 | 0·762 | (-292·2, 369·5) |
| Mother: hospital inpatient (non-delivery) costs | 216·2 (73·8) | 45·8 (29·0) | 170·4 | 0·062 | (18·8, 344·1) |
| Mother: A&E costs | 27·6 (9·3) | 310·7 (278·9) | -283·1 | 0·233 | (-1103·6, 10·6) |
| Mother: outpatient care costs | 50·0 (26·5) | 73 (34·9) | -23·0 | 0·595 | (-116·0, 55·1) |
| Mother: community care costs | 337·2 (52·8) | 409·5 (51·6) | -72·3 | 0·344 | (-210·0, 66·8) |
| Mother: medication costs | 121·6 (92·4) | 49·6 (27·7) | 72·0 | 0·520 | (-58·2, 298·7) |
| Mother: personal social service costs | 1·2 (0·9) | 106·1 (105·6) | -104·9 | 0·243 | (-400·8, 1·9) |
| Mother: legal service costs | 19·8 (10·5) | 17·8 (8·3) | 2·0 | 0·891 | (-23·7, 27·4) |
| Mother: other costs | 82·6 (24·4) | 92·9 (38·8) | -10·4 | 0·813 | (-119·0, 68·3) |
| Mother: total costs | 2778·8 (243·3) | 2977·3 (373·8) | -198·5 | 0·643 | (-1178·0, 563·8) |
|  |  |  |  |  |  |
| Baby: hospital inpatient care (readmission) costs | 1410·2 (1082·1) | 544·5 (209·4) | 865·7 | 0·503 | (-523·0, 4041·0) |
| Baby: A&E costs | 52·8 (12·8) | 45·4 (11·2) | 7·4 | 0·680 | (-21·2, 41·1) |
| Baby: outpatient care costs | 97·5 (50·6) | 41·7 (13·8) | 55·8 | 0·361 | (-16·3, 191·1) |
| Baby: community care costs | 512·0 (237·7) | 268·7 (28·1) | 243·3 | 0·389 | (-32·9, 846·5) |
| Baby: medication costs | 9·0 (3·1) | 4·2 (2·0) | 4·8 | 0·244 | (-2·1, 12·7) |
| Baby: other costs | 43·2 (30·7) | 13·2 (4·2) | 30·1 | 0·41 | (-10·3, 109·8) |
| Baby-total costs | 2124·7 (1130·0) | 917·7 (217·1) | 1207·0 | 0·371 | (-354·7, 4233·8) |
|  |  |  |  |  |  |
| **Total mother and baby costs** | **4903·5 (1183·8)** | **3895·0 (433·1)** | **1008·5** | **0·486** | **(-848·1, 4045·6)** |
| **2 to 6 months (n=136 total; n=81 intervention and n=55 control)** | | |  |  |  |
| Mother: hospital inpatient readmission costs | 0 (0) | 5·4 (5·3) | -5·4 | 0·226 | (-21·9, 0·0) |
| Mother: A&E costs | 10·2 (6·2) | 11·2 (6·3) | -1·1 | 0·908 | (-18·7, 15·8) |
| Mother: outpatient care costs | 7·6 (5·6) | 11·2 (8·2) | -3·6 | 0·711 | (-27·1, 13·7) |
| Mother: community care costs | 223·3 (132·4) | 95·9 (19·4) | 127·4 | 0·432 | (-31·7, 490·8) |
| Mother: medication costs | 7·0 (2·0) | 3·3 (1·1) | 3·7 | 0·160 | (-0·3, 8·4) |
| Mother: personal social service costs | 0 (0) | 0 (0) | 0 | N/A | N/A |
| Mother: legal service costs | 9·8 (5·0) | 27·3 (18·0) | -17·5 | 0·275 | (-68·3, 7·9) |
| Mother: other costs | 7·9 (3·0) | 3·6 (3·2) | 4·3 | 0·338 | (-6·1, 12·0) |
| Mother: total costs | 265·7 (134·0) | 157·9 (32·7) | 107·8 | 0·515 | (-70·3, 482·4) |
|  |  |  |  |  |  |
| Baby: hospital inpatient readmission costs | 287·0 (6·3) | 493·5 (95·0) | -206·5 | 0·010 | (-424·5, -54·2) |
| Baby: A&E costs | 73·8 (32·4) | 74·9 (18·0) | -1·2 | 0·978 | (-55·9, 90·8) |
| Baby: outpatient care costs | 63·3 (19·6) | 52·2 (23·9) | 11·1 | 0·720 | (-55·5, 72·4) |
| Baby: community health care costs | 138·4 (17·0) | 126·7 (19·5) | 11·6 | 0·658 | (-41·5, 65·9) |
| Baby: medication costs | 51·7 (44·3) | 19·5 (13·6) | 32·2 | 0·559 | (-27·5, 160·0) |
| Baby: other costs | 10·7 (4·2) | 22·3 (12·2) | -11·6 | 0·305 | (-41·2, 9·3) |
| Baby: total costs | 624·7 (69·9) | 789·1 (132·1) | -164·4 | 0·235 | (-482·1, 106·9) |
|  |  |  |  |  |  |
| **Total mother and baby costs** | **890·4 (151·4)** | **947·0 (142·0)** | **-56·6** | **0·795** | **(-450·2, 434·1)** |
| **6 to 12 months (n=138 total; n=81 intervention and n=57 control)** | | |  |  |  |
| Mother: hospital inpatient readmission costs | 25·6 (12·4) | 2·6 (2·6) | 23·0 | 0·127 | (4·3, 53·4) |
| Mother: A&E costs | 22·9 (8·9) | 7·2 (5·0) | 15·7 | 0·172 | (-4·5, 36·4) |
| Mother: outpatient care costs | 38·0 (14·9) | 104·3 (64·5) | -66·3 | 0·246 | (-230·9, 31·3) |
| Mother: community care costs | 121·1 (24·1) | 160·3 (40·8) | -39·2 | 0·381 | (-132·7, 46·7) |
| Mother: medication costs | 69·8 (56·1) | 19·1 (7·6) | 50·7 | 0·452 | (-14·8, 224·2) |
| Mother: personal social service costs | 0 (0) | 0 (0) | 0 | N/A | N/A |
| Mother: legal service costs | 23·4 (16·1) | 24·2 (14·5) | -0·8 | 0·972 | (-39·3, 50·3) |
| Mother: other costs | 39·6 (15·8) | 58·8 (36·6) | -19·2 | 0·595 | (-123·0, 35·5) |
| Mother: total costs | 340·4 (88·7) | 376·6 (105·5) | -36·2 | 0·793 | (-318·6, 207·2) |
|  |  |  |  |  |  |
| Baby: hospital inpatient readmission costs | 562·3 (136·2) | 848·8 (200·2) | -286·5 | 0·222 | (-764·5, 154·6) |
| Baby: A&E costs | 106·8 (20·8) | 86·7 (19·9) | 20·1 | 0·504 | (-33·2, 75·2) |
| Baby: outpatient care costs | 7·6 (7·6) | 50·4 (17·2) | -42·8 | 0·013 | (-79·1, -6·4) |
| Baby: community care costs | 123·3 (20·8) | 163·4 (21·2) | -40·1 | 0·192 | (-95·5, 21·7) |
| Baby: medication costs | 24·5 (8·5) | 130·0 (99·4) | -105·5 | 0·210 | (-377·3, 19·0) |
| Baby: other costs | 16·1 (7·1) | 54·0 (36·7) | -37·9 | 0·237 | (-152·7, 12·4) |
| Baby: total costs | 840·6 (161·1) | 1333·3 (261·9) | -492·7 | 0·093 | (-1120·3, 90·0) |
|  |  |  |  |  |  |
| **Total mother and baby costs** | **1181·0 (206·9)** | **1709·9 (288·5)** | **-528·9** | **0·128** | **(-1203·7, 130·8)** |
| **Entire follow-up period (n=129 total; n=77 intervention and n=52 control)** | | |  |  |  |
|  | | |  |  |  |
| Mother: delivery costs | 1945·0 (120·9) | 1846·2 (126·5) | 98·8 | 0·584 | (-211·6, 434·9) |
| Mother: hospital inpatient (non-delivery) costs | 257·2 (78·4) | 60·6 (38·0) | 196·6 | 0·053 | (42·3, 359·4) |
| Mother: A&E costs | 64·2 (14·9) | 364·5 (316·4) | -300·3 | 0·251 | (-1029·6, 40·5) |
| Mother: outpatient care costs | 98·5 (38·0) | 130·1 (73·1) | -31·6 | 0·678 | (-237·3, 99·5) |
| Mother: community care costs | 689·7 (161·5 ) | 665·9 (87·9) | 23·8 | 0·910 | (-288·2, 441·4) |
| Mother: medication costs | 209·9 (156·8 ) | 62·1 (30·8) | 147·8 | 0·445 | (-52·9, 555·1) |
| Mother: personal social service costs | 1·2 (1·0) | 120·4 (119·8) | -119·1 | 0·228 | (-488·5, 2·0) |
| Mother: legal service costs | 46·2 (18·9) | 71·7 (33·4) | -25·6 | 0·477 | (-115·4, 41·7) |
| Mother: other costs | 137·9 (32·8) | 149·6 (60·8) | -11·7 | 0·855 | (-163·9, 108·8) |
| Mother: total costs | 3449·8 (355·1) | 3471·0 (436·7) | -21·1 | 0·970 | (-1107·7, 1016·3) |
|  |  |  |  |  |  |
| Baby: hospital inpatient readmission costs | 2313·7 (1186·2) | 1747·8 (300·5) | 565·9 | 0·700 | (-1070·9, 3518·0) |
| Baby: A&E costs | 232·8 (44·5) | 210·0 (37·7) | 22·8 | 0·716 | (-85·9, 137·6) |
| Baby: outpatient care costs | 175·7 (63·4) | 145·9 (44·8) | 29·8 | 0·728 | (-111·5, 198·3) |
| Baby: community care costs | 765·4 (256·4) | 550·3 (53·6) | 215·1 | 0·497 | (-122·1, 926·3) |
| Baby: medication costs | 87·4 (47·2) | 162·7 (109·5) | -75·3 | 0·481 | (-371·0, 99·6) |
| Baby: other costs | 72·7 (36·9) | 74·6 (41·5) | -2·0 | 0·972 | (-113·5, 95·1) |
| Baby: total costs | 3647·7 (1305·9) | 2891·3 (383·5) | 756·3 | 0·642 | (-1074·7, 4555·7) |
|  |  |  |  |  |  |
| **Total mother and baby costs** | **7097·5 (1416·8)** | **6362·3 (631·0)** | **735·2** | **0·684** | **(-1670·7, 4762·3)** |
| **gFNP intervention costs** | **2036·0 (306·9)** | **0 (0)** | **2036·0** | **<0·0001** | **(1501·3, 2709·6)** |
| **Total NHS and PSS costs**  **(including intervention)** | **8876·6 (1399·0)** | **6066·4 (601·0)** | **2810·3** | **0·069** | **(337·8, 6607·1)** |
| **Total societal costs**  **(including intervention)** | **9133·5 (1435·4)** | **6362·3 (631·0)** | **2771·2** | **0·077** | **(685·4, 6865·4)** |

SE denotes standard error; CI denotes confidence interval ; A&E denotes accident and emergency ; PSS denotes personal social services ; N/A denotes not applicable.

^a^ P value calculated using Student t test, 2 tail unequal variance.

^b^ Non-parametric bootstrap estimation using 10,000 replications, bias corrected.

**Table A4: Cost-effectiveness results based upon the QALY and maltreatment outcomes: Imputed data, societal perspective (£, 2014**–**2015 prices)**

| **Outcome Measure** | **Mean costs (95% CI)** | | |  | **Mean effects (95% CI)** | | |  |  | **Probability gFNP intervention is** | | | |  |
| --- | --- | --- | --- | --- | --- | --- | --- | --- | --- | --- | --- | --- | --- | --- |
|  | Intervention  (£) | Control  (£) | Difference  (£) |  | Intervention | Control | Difference |  | ICER (£) | **More effective^*^ (%)** | **Less costly^*^ (%)** | **Cost-effective^*^**  **(%)^#^** | **Cost-effective^*^**  **(%)^±^** | **Cost-effective^*^**  **(%)^∞^** |
| **QALY** | N=97 | N=67 |  |  | N=97 | N=67 |  |  |  |  |  |  |  |  |
|  | 9074  (6206,  11942) | 6279 (5112, 7445) | 2795  (-277,  5867) |  | 0·92  (0·90,  0·94) | 0·93  (0·91,  0·94) | -0·01  (-0·03,  0·02) |  | -333775  (NW) | 19·2 | 3·2 | 2·6 | 2·5 | 2·9 |
| **AAPI** | N=97 | N=67 |  |  | N=97 | N=67 |  |  |  |  |  |  |  |  |
|  | 9074  (6206,  11942) | 6279 (5095, 7463) | 2795  (-302,  5892) |  | 0·27  (0·14,  0·40) | 0·25  (0·12,  0·38) | 0·02  (-0·17,  0·21) |  | 150152  (NE) | 58·4 | 1·3 | 14·1 | 19·0 | 28·2 |
| **CARE Index (maternal sensitivity domain)** | N=97 | N=67 |  |  | N=97 | N=67 |  |  |  |  |  |  |  |  |
|  | 9074  (6206,  11942) | 6279 (5095, 7463) | 2795  (-302,  5892) |  | 3·97  (3·54,  4·39) | 4·84  (4·30,  5·38) | -0·87  (-1·55,  -0·19) |  | -3212  (NW) | 1·2 | 1·0 | <1 | <1 | <1 |
| The gFNP intervention was considered to be “cost-effective” if it had positive net benefit at a: **^#^**GBP £15,000 cost-effectiveness threshold, **^±^**GBP £20,000 cost-effectiveness threshold, **^∞^**GBP £30,000 cost-effectiveness threshold  ^*^ Based on 10,000 bootstrap replicates of the dataset.  CI, confidence interval; ICER, incremental cost-effectiveness ratio; NW, north-west quadrant of cost-effectiveness plane· NE, north-east quadrant of the cost-effectiveness plane. | | | | | | | | | | | | | | |

**Table A5: Cost-effectiveness based upon QALY and maltreatment outcomes: Complete case analysis, NHS & PSS perspective (£,2014**–**2015 prices)**

| **Outcome Measure** | **Mean costs (95% CI)** | | | **Mean effects (95% CI)** | | |  | **Probability gFNP intervention is** | | | |  |
| --- | --- | --- | --- | --- | --- | --- | --- | --- | --- | --- | --- | --- |
|  | Intervention  (£) | Control  (£) | Difference  (£) | Intervention | Control | Difference | ICER (£) | **More effective^*^ (%)** | **Less costly^*^ (%)** | **Cost-effective^*^**  **(%)^#^** | **Cost-effective^*^**  **(%)^±^** | **Cost-effective^*^**  **(%)^∞^** |
| **QALY** | N=56 | N=45 |  | N=56 | N=45 |  |  |  |  |  |  |  |
|  | 9085  (6061, 12109) | 6005 (4788,  7222) | 3080  (-102,  6262) | 0·84  (0·81,  0·88) | 0·86  (0·81,  0·90) | -0·01  (-0·07, 0·05) | -217674  (NW) | 32·7 | 1·1 | 1·9 | 2·0 | 2·5 |
| **AAPI** | N=59 | N=49 |  | N=59 | N=49 |  |  |  |  |  |  |  |
|  | 9085  (5654,  12516) | 6005  (4776,  7234) | 3080  (-594,  6754) | 0·25  (0·08,  0·42) | 0·15  (0·01,  0·29) | 0·10  (-0·13,  0·33) | 30843  (NE) | 77·8 | 2·4 | 28·7 | 37·0 | 47·4 |
| **CARE Index (maternal sensitivity domain)** | N=52 | N=41 |  | N=52 | N=41 |  |  |  |  |  |  |  |
|  | 9085  (7283,  10887) | 6005  (4706,  7304) | 3080  (885,  5275) | 4·06  (3·47,  4·65) | 4·66  (3·90,  5·42) | -0·60  (-1·55,  0·35) | -5126  (NW) | 12·2 | 2·8 | 6·6 | 8·0 | 9·0 |

The gFNP intervention was considered to be “cost-effective” if it had positive net benefit at a: **^#^**GBP £15,000 cost-effectiveness threshold, **^±^**GBP £20,000 cost-effectiveness threshold, **^∞^**GBP £30,000 cost-effectiveness threshold

^*^ Based on 10,000 bootstrap replicates of the dataset. CI, confidence interval; ICER, incremental cost-effectiveness ratio; NW, north-west quadrant of cost-effectiveness plane; NE, north-east quadrant of the cost-effectiveness plane.

**Table A6: Sensitivity analysis that varied gFNP session attendance and group size: Complete case analysis, NHS and PSS perspective (£, 2014**–**2015 prices)**

| **Sensitivity analysis** | **Mean costs (95% CI)** | | | **Mean effects (QALYs) (95% CI)** | | |  | **Probability gFNP intervention is** | | | |  |
| --- | --- | --- | --- | --- | --- | --- | --- | --- | --- | --- | --- | --- |
|  | Intervention  (£) | Control  (£) | Difference  (£) | Intervention | Control | Difference | ICER (£) | **More effective^*^ (%)** | **Less costly^*^ (%)** | **Cost-effective^*^**  **(%)^#^** | **Cost-effective^*^**  **(%)^±^** | **Cost-effective^*^**  **(%)^∞^** |
| **Higher mean no. of sessions** | N=56 | N=45 |  | N=56 | N=45 |  |  |  |  |  |  |  |
|  | 7389  (5137,  9640) | 6120  (5164,  7076) | 1269  (-1184,  3721) | 0·92  (0·90,  0·94) | 0·93  (0·91,  0·94) | -0·01  (-0·03,  0·02) | -151502 (NW) | 19·2 | 28·8 | 21·5 | 20·1 | 18·4 |
| **Lower mean no. of sessions** | N=56 | N=45 |  | N=56 | N=45 |  |  |  |  |  |  |  |
|  | 128273  (90388,  166157) | 6264  (5239,  7290) | 122008  (84104,  159913) | 0·92  (0·90,  0·94) | 0·93  (0·91,  0·94) | -0·01  (-0·03,  0·02) | -14600000  (NW) | 19·2 | <1 | <1 | <1 | <1 |
| **Higher no. of women per group** | N=56 | N=45 |  | N=56 | N=45 |  |  |  |  |  |  |  |
|  | 7668  (5424,  9913) | 6117  (5180,  7053) | 1552  (-882,  3985) | 0·92  (0·90,  0·94) | 0·93  (0·91,  0·94) | -0·01  (-0·03,  0·02) | -185300  (NW) | 19·2 | 13·9 | 10·2 | 9·7 | 9·7 |
| **Lower no. of women per group** | N=56 | N=45 |  | N=56 | N=45 |  |  |  |  |  |  |  |
|  | 12679  (9829,  15529) | 6129  (5190,  7069) | 6550  (3557,  9543) | 0·92  (0·90,  0·94) | 0·93  (0·91,  0·94) | -0·01  (-0·03,  0·02) | -782195  (NW) | 19·2 | <1 | <1 | <1 | <1 |

The gFNP intervention was considered to be “cost-effective” if it had positive net benefit at a: **^#^**GBP £15,000 cost-effectiveness threshold, **^±^**GBP £20,000 cost-effectiveness threshold, **^∞^**GBP £30,000 cost-effectiveness threshold. ^*^ Based on 10,000 bootstrap replicates of the dataset.

CI, confidence interval; ICER, incremental cost-effectiveness ratio; NW, north-west quadrant of cost-effectiveness plane.

**Table A7: Sub-group analyses: Incremental cost-effectiveness of gFNP intervention in pre-specified sub-groups (£, 2014**–**2015 prices)**

| **Sensitivity analysis** | **Mean costs (95% CI)** | | | **Mean effects (QALYs) (95% CI)** | | |  | **Probability gFNP intervention is** | | | |  |
| --- | --- | --- | --- | --- | --- | --- | --- | --- | --- | --- | --- | --- |
|  | Intervention  (£) | Control  (£) | Difference  (£) | Intervention | Control | Difference | ICER (£) | **More effective^*^ (%)** | **Less costly^*^ (%)** | **Cost-effective^*^**  **(%)^#^** | **Cost-effective^*^**  **(%)^±^** | **Cost-effective^*^**  **(%)^∞^** |
| **Program completers** | N=29 | N=67 |  | N=29 | N=67 |  |  |  |  |  |  |  |
|  | 9863  (7552,  12174) | 6107  (5170,  7043) | 3757  (1283,  6230) | 0·90  (0·87,  0·93) | 0·93  (0·91,  0·94) | -0·03  (-0·06,  0·01) | -140686  (NW) | 5·3 | <1 | <1 | <1 | <1 |
| **Program non-completers** | N=68 | N=67 |  | N=68 | N=67 |  |  |  |  |  |  |  |
|  | 7449  (4428,  10470) | 6107  (5170,  7043) | 1342  (-1817,  4502) | 0·93  (0·91,  0·95) | 0·93  (0·91,  0·94) | -0·001  (-0·03,  0·02) | -1122691  (NW) | 45·9 | 20·9 | 20·9 | 20·9 | 20·6 |
| **Program phase one** | N=27 | N=67 |  | N=27 | N=67 |  |  |  |  |  |  |  |
|  | 7771  (5358,  10184) | 6107  (5170,  7043) | 1665  (-872,  4201) | 0·91  (0·88,  0·95) | 0·93  (0·91,  0·94) | -0·01  (-0·06,  0·03) | -125764  (NW) | 22·1 | 8·3 | 5·7 | 5·7 | 5·4 |
| **Program phases two and three** | N=44 | N=67 |  | N=44 | N=67 |  |  |  |  |  |  |  |
|  | 9783  (7446,  12120) | 6107  (5105,  7108) | 3677  (1146,  6207) | 0·91  (0·88,  0·94) | 0·93  (0·90,  0·95) | -0·02  (-0·06,  0·02) | -217390  (NW) | 34·0 | 2·1 | 1·3 | 1·3 | 1·2 |

The gFNP intervention was considered to be “cost-effective” if it had positive net benefit at a: **^#^**GBP £15,000 cost-effectiveness threshold, **^±^**GBP £20,000 cost-effectiveness threshold, **^∞^**GBP £30,000 cost-effectiveness threshold

^*^ Based on 10,000 bootstrap replicates of the dataset.

CI, confidence interval; ICER, incremental cost-effectiveness ratio; NW, north-west quadrant of cost-effectiveness plane.
